# Combining machine learning, crowdsourcing and expert knowledge to detect chemical-induced diseases in text

**DOI:** 10.1093/database/baw094

**Published:** 2016-06-15

**Authors:** Àlex Bravo, Tong Shu Li, Andrew I. Su, Benjamin M. Good, Laura I. Furlong

**Affiliations:** ^1^Research Programme on Biomedical Informatics (GRIB), IMIM, UPF, Barcelona, Spain and; ^2^Department of Molecular and Experimental Medicine, the Scripps Research Institute, La Jolla, CA, USA

## Abstract

Drug toxicity is a major concern for both regulatory agencies and the pharmaceutical industry. In this context, text-mining methods for the identification of drug side effects from free text are key for the development of up-to-date knowledge sources on drug adverse reactions. We present a new system for identification of drug side effects from the literature that combines three approaches: machine learning, rule- and knowledge-based approaches. This system has been developed to address the Task 3.B of Biocreative V challenge (BC5) dealing with Chemical-induced Disease (CID) relations. The first two approaches focus on identifying relations at the sentence-level, while the knowledge-based approach is applied both at sentence and abstract levels. The machine learning method is based on the BeFree system using two corpora as training data: the annotated data provided by the CID task organizers and a new CID corpus developed by crowdsourcing. Different combinations of results from the three strategies were selected for each run of the challenge. In the final evaluation setting, the system achieved the highest Recall of the challenge (63%). By performing an error analysis, we identified the main causes of misclassifications and areas for improving of our system, and highlighted the need of consistent gold standard data sets for advancing the state of the art in text mining of drug side effects.

**Database URL**: https://zenodo.org/record/29887?ln¼en#.VsL3yDLWR_V

## Introduction

Text mining systems help us to extract, structure, integrate and automatically analyze information contained in millions of biomedical publications. Identifying the named entities of interest (Named Entity Recognition or NER) and extracting their relationships (Relation Extraction or RE) are still some of the most important challenges for biomedical text mining. The potential value of text mining approaches focused on detection of drugs and their relation with disease is evident in areas such as drug safety monitoring or prediction of drug toxicity.

The BC5 challenge designed specific tasks involving the identification of diseases and chemicals, and the extraction of Chemical-induced Disease (CID) relations to promote the development of text mining solutions for the study of drug side effects (SEs). Previous work on text mining aimed at identifying drug and their relations with diseases applied a variety of strategies, including co-occurrence based statistics (1, 2), patterns (3) and machine learning approaches (4). Xu and Wang (5) applied a pattern-learning approach to identify drug treatments from MEDLINE with high precision. Gurulingappa *et al.* used the JSRE system (6), originally developed to identify interactions between proteins, for the identification of drug SEs from medical case reports, achieving 87% *F*-score in the ADE corpus. Bravo *et al.* (17) developed a system based also in JSRE to identify drug-disease relations, introducing a new kernel using dependency trees information. This system achieved 79.3% *F*-score by 10-fold cross-validation in the EU-ADR corpus. JReX, a system for event extraction originally developed for protein interactions, has also been adapted to the domain of pharmacogenomics (7). By leveraging on syntactic and semantic information of the sentence, JReX achieved 79% *F*-score in the identification of drug treatment relations. Other authors have proposed approaches based on previous knowledge of drug adverse reactions as an alternative to machine learning-based approaches, which can be difficult to develop due to their need of large training datasets (8). For instance, Kang et al developed a method based on the recognition of concepts followed by the detection of associations using a database on drug SEs derived from the UMLS (8). In addition to scientific publications, clinical records and public forums are relevant resources to detect drug adverse reactions (9–12).

Remarkably, most of the previous work restricted the search of drug adverse reactions to relations between a drug and a disease co-occurring in the same sentence. In this regard, the BC5 CID task constitutes a new challenge since the relations are annotated both at the sentence and at the whole-document level (e.g. span several sentences).

In this article, we present a system to identify CID-relations for the BC5 task (13) that combines several of the approaches previously proposed for the identification of relations between drugs and diseases, namely patterns, machine learning approach and the use of background knowledge. In addition, to showcase the application of crowdsourcing as a suitable approach to develop training data for text mining, we have developed a new corpus on CID-relations using a crowdsourcing approach to train the machine learning system. We present the results obtained on the BC5 development and evaluation sets (BC5_D_ and BC5_E_), and the results of the error analysis performed on the results of our system.

## Methods

**Figure 1.**
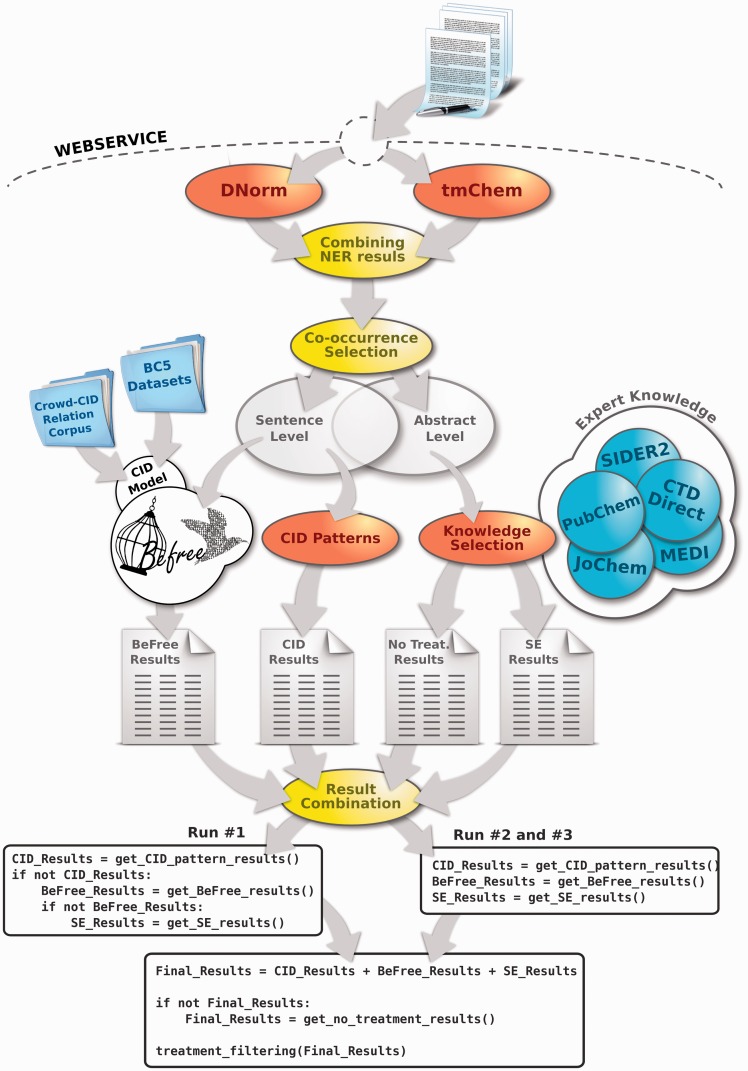
The workflow diagram of the developed system for CID-RE. Note that the CID-relations found both at abstract and sentence level are processed by BeFree, CID-patterns and EK-based approach.

The task 3.B of Biocreative V (BC5) focuses on CID relations found in Medline abstracts. These relations can be expressed in a single sentence or span multiple sentences. We developed a system to identify CID-relations both at the sentence and the abstract level, combining three strategies: machine learning, pattern-matching and a knowledge-based approach. The first two approaches are aimed at identifying relations at the sentence-level, while the knowledge-based approach is applied at both sentence and abstract levels. Figure 1 shows a scheme of the system and the three configurations used for the challenge runs.

The NER systems DNorm (14, 15) and tmChem (16) were used to identify disease and chemical mentions, respectively, in the documents. In order to homogenize our results with other works, the NER results of DNorm and tmChem provided by the BC5 organizers were used for the evaluation of the BC5_E_ results.

The NER results were post-processed to improve the identification of relationships for the CID task. During the development phase, we detected some False Positive (FP) predictions at the RE level that were originated by errors in the previous NER step. In the case of diseases, the identification by the NER of the disease that a patient is suffering and is the therapeutic indication of the drug could later.

Lead to a FP prediction of our system. An example of this type of sentences is the following:

- ‘Prolonged left ventricular dysfunction occurs in ‘patients with coronary artery disease’ after both dobutamine and exercise induced myocardial ischaemia’ (PMID: 10677406).
- ‘Risk of transient hyperammonemic encephalopathy in ‘cancer patients’ who received continuous infusion of 5-fluorouracil with the complication of dehydration and infection’ (PMID: 10327032).

In these cases, we applied regular expressions (such as ‘DISEASE patient(s)’) to filter out the diseases that are not actually SEs of the drug in the context of the sentence.

For the chemicals, we observed that some simple chemicals such as calcium (Ca), carbon (C) and oxygen (O) were leading to FP predictions at the RE and were therefore removed from the annotations.

In addition, when the chemical and the disease entities overlapped in the text, we applied a set of simple rules (such as to detect the longest mention or determine if a mention is inside another mention) for the selection of the correct entity.

Then, the NER results were processed to extract co-occurrences at the sentence level. The abstracts were splitted in sentences, and each offset corresponding to a mention at the abstract-level was converted to sentence-level offset.

### Machine learning approach

The machine learning approach is based on the BeFree system (17), a tool to identify relations between biomedical entities in free text. BeFree is composed of a module for Biomedical NER and a module for RE based on Support Vector Machines (SVM). In this work, only the RE module was used. The RE module uses different morpho-syntactic features which are extracted for each sentence. The methods to obtain such features from a text can be classified in two groups: shallow and deep linguistic parsing. Due to the processing time constraints of the BC5 challenge, only shallow linguistic features were used (such as part-of-speech, lemma, and stem). A predictive model for CID-relations at the sentence level was developed using two different corpora: the annotation data provided by the CID task organizers (training and/or development sets) and a newly developed CID corpus (Crowd-CID relation corpus).

### Annotated data provided by BC5

All the abstracts from the training and development (BC5_T_ and BC5_D_) sets (18) were processed by the DNorm and tmChem NERs to identify disease and chemical entities. Then, the sentences including at least one co-occurrence between a disease and a chemical were selected as possible CID-relations for training the BeFree RE module. If the identifiers of a specific co-occurrence were reported as true by the gold standard (GS), the sentence and the CID pair was saved as a true CID-relation, otherwise it was considered a false CID-relation. According to this procedure, 3,632 true and 6,122 false CID-relations were found in 3,728 sentences. The BeFree model trained on this set achieved a performance of 59.86, 68.82 and 63.96% of precision, recall and *F*-score, respectively, by 10-fold cross-validation.

### Crowd-CID relation corpus

A set of 1,990,686 abstracts focused on diseases, treatments and SEs were retrieved from PubMed. Then, 3,000 abstracts were randomly selected for the annotation process. The DNorm and the tmChem NERs were used to identify disease and chemical mentions and sentences containing at least one co-occurrence between a disease and a chemical name were selected for annotation by crowd workers. Overall, a set of 2,756 PubMed abstracts contained a total of 17,198 unique sentences with at least one chemical or disease annotation. Of these 17,198 sentences, only 2,953 sentences contained both a chemical and a disease annotation. There were a total of 3,068 unique chemical-disease identifier pairs that resulted in 5,160 unique sentence-concept-pair triples representing crowd verification tasks.

Workers on the Crowdflower microtask platform were presented each sentence containing a putative CID-relation and asked to judge whether the relation held. A total of 290 workers were provided detailed instructions with multiple examples and were required to pass a quiz with 70% minimum accuracy before gaining access to the task, where 134 workers (46.2%) passed. In addition, the system removed results from workers who failed hidden test questions embedded in the task stream when they dropped below 70% accuracy. Five workers processed each sentence and were paid $0.02 per sentence completed. The tasks were completed within 7 h and cost a total of $763.92.

For the 5160 verification tasks, the distribution of the crowd response was overwhelmingly that there was no relation between the presented chemical and disease. Only 13.5% of the tasks received three or more votes as being true CID-relations, while 64.0% of the tasks received full agreement between workers for being false CID-relations. Although the percentage of CID-relations was low, these figures are similar to what would be expected based on the pure co-occurrence model, especially considering that only sentence-bound relations were curated for BeFree.

Worker judgments were aggregated based on answer choice, resulting in the number of workers who said a relation was true, using majority rules. We set a threshold of three or more votes for answer choice to select the final corpus, which resulted in 698 true and 4,462 false CID relations.

For training the BeFree system, the corpus was balanced by selecting at random a subset of false examples, obtaining a total of 1,628 examples from 1,251 sentences (698 true and 930 false examples).

The BeFree model trained with this subset of crowd-CID relation corpus achieved a performance of 82.03, 73.39 and 76.82% of Precision, Recall and *F*-score, respectively, by 10-fold cross-validation. The CID-crowd relation corpus is available at https://zenodo.org/record/29887?ln=en#.VsL3yDLWR_V.

### Rule-based approach

The rule-based approach is the most straightforward technique to identify relations between two entities at the sentence level. Xu and Wang 2014 reported the most frequent patterns used to express chemical-SE relations in FDA drug labels (3). We used a subset of these patterns (such as ‘CHEMICAL-induced SE’, ‘CHEMICAL-associated SE’, ‘SE caused by CHEMICAL’ and ‘SE during CHEMICAL’) to identify CID-relations at the single sentence level.

### Expert knowledge-based approach

We checked if a CID-relation identified in the text (at the sentence or abstract level) was already known as a drug SE, or as the therapeutic indication of the drug. In particular, we removed those CID-relations identified at the abstract level that were annotated as the therapeutic indication in our knowledge base. This knowledge base was developed by integrating information from different resources (CTD (19), SIDER2 (20) and MEDI (21) databases) that contain information on drug therapeutic indications and SEs. JoChem (22) and PubChem (23) were used to map the chemical entities to MeSH identifiers. The UMLS Metathesurus MRCONSO table was used to map between MeSH and UMLS identifiers for the diseases. The database contains 28,455 chemical-disease associations labeled as therapeutic, and 55,960 labelled as SEs. Associations labelled both as therapeutic and SEs were not included in the database.

### Combined system

Figure 1 shows a schema of our combined system to detect CID-relations for the BC5 task. The CID-relations stated at the sentence level (that could also be stated at the abstract level) were processed by the CID-patterns approach and BeFree, while the CID-relations that were found at the abstract level were processed by the knowledge-based approach to identify known side-effect relations (SE). Note that the set of CID-relations processed by the knowledge-based approach include CID-relations that could be stated at both abstract and sentence-level. The BeFree model was trained with the crowd-CID relation corpus and the BC5_T_ set when assessing the system performance on the development set. For the challenge evaluation set, the model was trained on the crowd-CID relation corpus and the BC5_T_ and BC5_D_ sets. Finally, after obtaining the union of the results of each approach, we removed those relations that are known as therapeutic indication of the drug as a final filtering step (represented in the last box of Figure 1 as ‘treatment_filtering(Final_Results)’).

## Results and Discussion

We present the results obtained on the BC5_D_ and the BC5_E_ sets. Different configurations of the system were evaluated on 50 abstracts from the BC5_D_ set during the development phase. The configurations achieving better *F*-score were selected for the three runs on the BC5_E_ set.

### Evaluation on the BC5_D_ and BC5_E_ sets

From the 1,012 CID-relations present in the BC5_D_, ∼70% were found at the single sentence level. On the other hand, 70% of CID-relations were annotated as true associations. Table 1 shows the results obtained with our system. Applying a simple co-occurrence approach on the chemical and disease mentions identified by the provided BC5 NER systems on this GS results in 16.43% Precision and 76.45% Recall, setting the upper boundary for Recall in the development set (16.46% Precision and 81.71% Recall were obtained on the subset of 50 abstracts from BC5_D_).

**Table 1.** Performance of the different methods evaluated on the BC5_D_ (the first 50 abstracts)

Exp.	Method	Train data	Level	*R*	*P*	*F*
1	Co-occurrence	NA	Both	81.71	16.46	27.40
2	EK	NA	Abst.	60.98	42.37	50.00
3	CID-patterns	NA	Sent.	12.20	71.43	20.83
4	CID-pat. + EK	NA	Both	63.41	42.62	50.98
5	BeFree system	BC5_T_	Sent.	47.56	38.61	42.62
6	BeFree system	crowdCID	Sent.	54.88	33.33	41.47
7	BeFree system	BC5_T_ + crowdCID	Sent.	53.66	39.20	45.62
8	Run no. 1	BC5_T_	Both	57.31	63.51	60.25
9	Run no. 1	crowdCID	Both	58.53	66.66	62.33
10	Run no. 1	BC5_T_ + crowdCID	Both	57.32	66.20	61.44
11	Run no. 1	BC5_T_ + crowdCID	Both	46.74	56.04	50.97
12^a^	Run no. 1	BC5_T+D_ + crowdCID	Both	38.27	48.57	42.81
13	Run no. 2	BC5_T_ + crowdCID	Both	79.27	43.91	56.52
14	Run no. 3	BC5_T_ + crowdCID	Both	79.27	44.83	57.27

a In this case, the full set (500 abstracts) was used.

By applying the expert knowledge (EK)-based approach to identify CID-relations at the abstract-level, we achieved 60.8, 42.37 and 50.00% Recall, Precision and an *F*-score, respectively. The low Precision obtained with a knowledge-based approach is surprising. Without performing an error analysis on the development data we can speculate that issues related with identifier mapping between the knowledge base and the evaluation data, or different annotation criteria between the knowledge bases and the evaluation set might result in a large number of FP. The low Recall can be explained by (i) not considering associations at sentence-level, (ii) limitations of the knowledge sources considered and (iii) issues related with identifier mapping.

On the other hand, the approaches aimed at detecting CID-relations at the sentence-level were also individually evaluated. The rule-based approach resulted in a high precision and low recall (71.43 and 12.20%, respectively), while the BeFree system achieved 39.20, 53.66 and 45.62 of precision, recall and *F*-score, respectively (38.61% of recall, 47.56% of precision and 45.62% of *F*-score were the results obtained only using the BC5_T_ set).

The different approaches were combined in 3 different ways for the challenge (Fig. 1). For the Run no. 1, the CID pattern approach was applied first, followed by BeFree and finally the knowledge-based approach. If a CID was found by the pattern based approach, the other two methods were not applied and the results reported were the ones resulting from pattern matching. In contrast, the three approaches were applied simultaneously in Run nos. 2 and 3, and the results were the union of the results of each of them. The difference between these two runs is that in Run no. 3 the CID pattern approach is only applied to the title of the abstract and not to the remaining text. In the three runs, the final set of results was filtered by removing those CID-relations that were annotated as therapeutic in our knowledge base.

Finally, the different approaches and the combination between them were also evaluated against the full BC5_E_ set. Table 2 shows the different results obtained in each experiment on the BC5_E_ set. Although the Precision and Recall values are lower than on the BC5_D_ set, they followed the same behavior.

**Table 2.** Performance of the different methods evaluated on the BC5_E_ (with NER results as provided by organizers)

Exp.	Method	Train data	Level	*R*	*P*	*F*
1	Co-occurrence	NA	Both	72.05	16.38	26.69
2	EK	NA	Abst.	56.10	39.19	46.14
3	CID-patterns	NA	Sent.	15.85	73.16	26.06
4	CID-pat. + EK	NA	Both	14.54	79.49	24.58
5	BeFree system	BC5_T+D_	Sent.	43.34	42.82	43.08
6	BeFree system	crowdCID	Sent.	45.59	36.05	40.27
7	BeFree system	BC5_T+D_ + crowdCID	Sent.	44.00	41.18	42.54
12	Run no. 1	BC5_T+D_ + crowdCID	Both	40.80	49.38	44.68
13	Run no. 2	BC5_T+D_ + crowdCID	Both	63.04	38.23	47.59
14	Run no. 3	BC5_T+D_ + crowdCID	Both	62.48	38.12	47.35

In contrast to the performance on BC5_D_, the best performance obtained on the BC5_E_ set was with Run no. 2, achieving 63.04% Precision, 38.23% Recall and 47.59% *F*-score. When compared with the performance on 50 documents of the BC5_D,_ the performance on the BC5_E_ set is significantly decreased. However, the results are not that different to the ones obtained on the 500 documents of the BC5_D_. Based on these results and those from comparisons between the 50 and 500 document set for Run nos. 2 and 3, we can suggest that the differences in performance arise from distinct sub-sets within the data that have different characteristics. Another factor explaining the differences would be the different data used to train the BeFree model in these two settings (BC5_D_ and BC5_E_ sets).

### Analysis of performance against the GS

**Figure 2.**
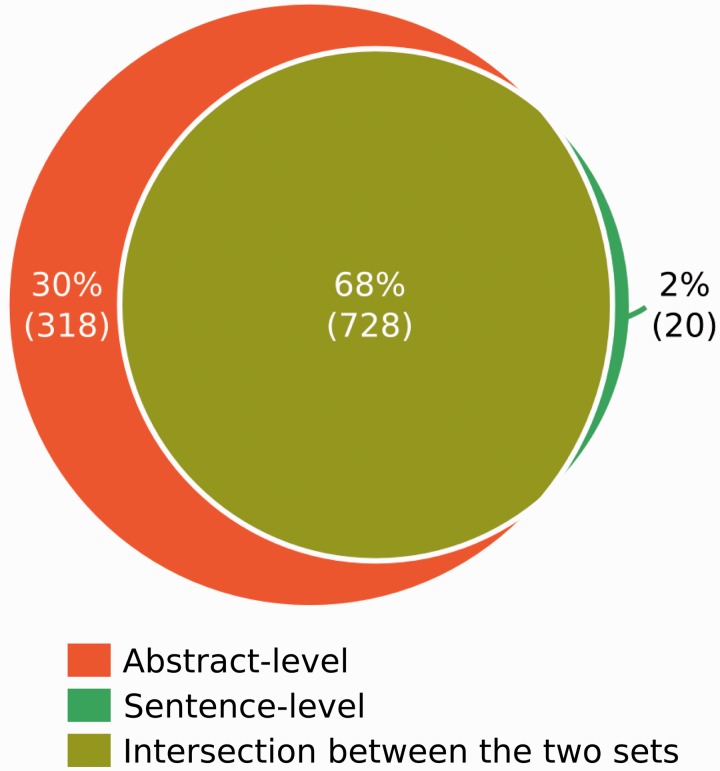
Number of Gold Standard CID associations stated in a single sentence (Sentence-level) or spanning several sentences (Abstract-level).

When the BC5_E_ set was provided by the BC5 organizers, several analysis were carried out comparing the GS annotations and the results of our system, in order to identify the causes of erroneous predictions produced by our system. The GD is composed of 1,066 CID-relations, where 30% are only stated spanning several sentences (abstract level), 2% are stated only at the sentence-level, and 68% are observed both at the abstract and the sentence levels (Figure 2). The different approaches that are the basis of our system were separately evaluated against the GS at the sentence, abstract and both levels.

**Figure 3.**
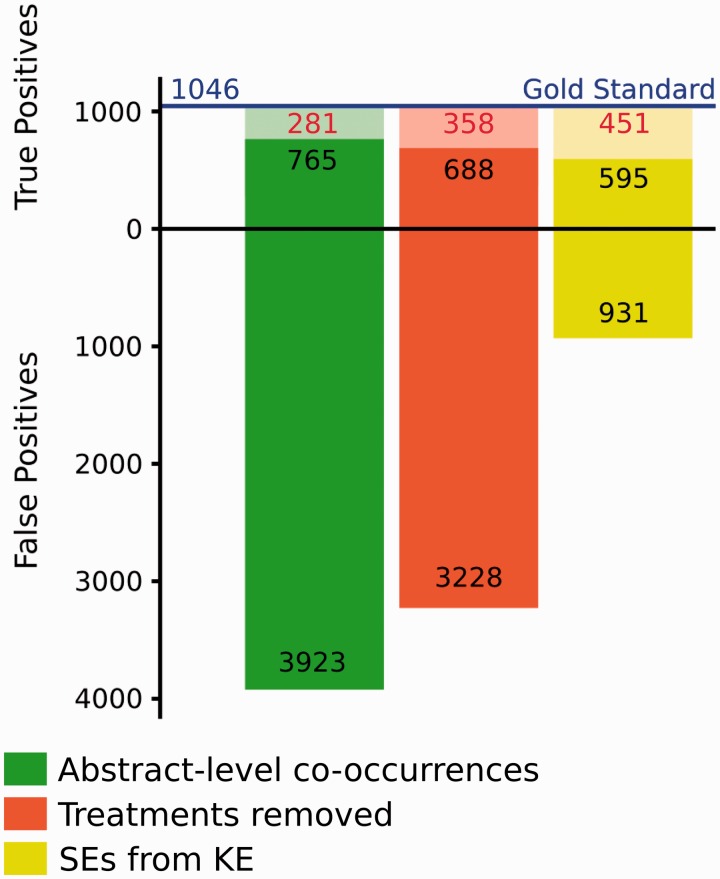
Number of CID-relations at the abstract level identified by the EK approach in relation to the Gold standard. In each column, the lighter colors represent the fraction of False Negatives (FN).

Since the EK approach aims at identifying CID-relations at the abstract-level, we used the subset of CID-relations stated at the abstract level (1,046) to analyse in detail its performance. Figure 3 shows the results obtained when applying a co-occurrence based approach and filtering the found drug-disease co-occurrences with information from a knowledge base. By simply considering all the possible co-occurrences of a drug and a disease at the abstract level, we correctly identify 765 CID-relations (TP, leading to a Recall of 73%) and miss 281 CID-relations (FN). The FNs are mainly due to errors in the NER. This approach produces, as expected, a large fraction of FP CID-relations (3,923). The number of FP can be decreased by ∼18% by considering background knowledge, in particular by removing those associations annotated as the therapeutic indication of the drug in our database. This filtering step also reduces the number of TP detected. This may due to the fact that some adverse events are related to the therapeutic effect of the drug. An example of these are the drugs prescribed to lower blood pressure, that can produce fainting as undesired effect when taken at high doses (24).

When applying an additional filtering step, such as selecting only those chemical-disease pairs annotated as SE associations in the knowledge base, we observe an important decrease of FP (∼76%), and also an increase in FNs. In both cases in which we apply background knowledge, the reduction of FP and TP results was due to the reduction of the overall number of results. Many TP results were discarded because their CID-relations were not found in the knowledge base, which can be explained by its incompleteness, or otherwise issues related with identifier mapping between the database and the evaluation data. In addition, not all FPs were removed, because the EK approach took all possible chemical–disease pairs detected in each abstract without considering the topic of the abstract or if the chemical–disease pairs were semantically linked. The presence of FP even after filtering with known CID-relations can be explained by: i) different criteria in the annotation of CID-relations for the BC5 and drug SE in the databases SIDER and CTD; ii) errors in the SE databases (in fact SIDER is developed using a text mining approach); iii) again, issues of identifier mappings for both drugs and diseases (for instance, UMLS identifiers are mainly used in these databases to describe a disease). We foresee that a high quality database on drug therapeutic indications and drug SEs could improve the results obtained with this kind of approach.

**Figure 4.**
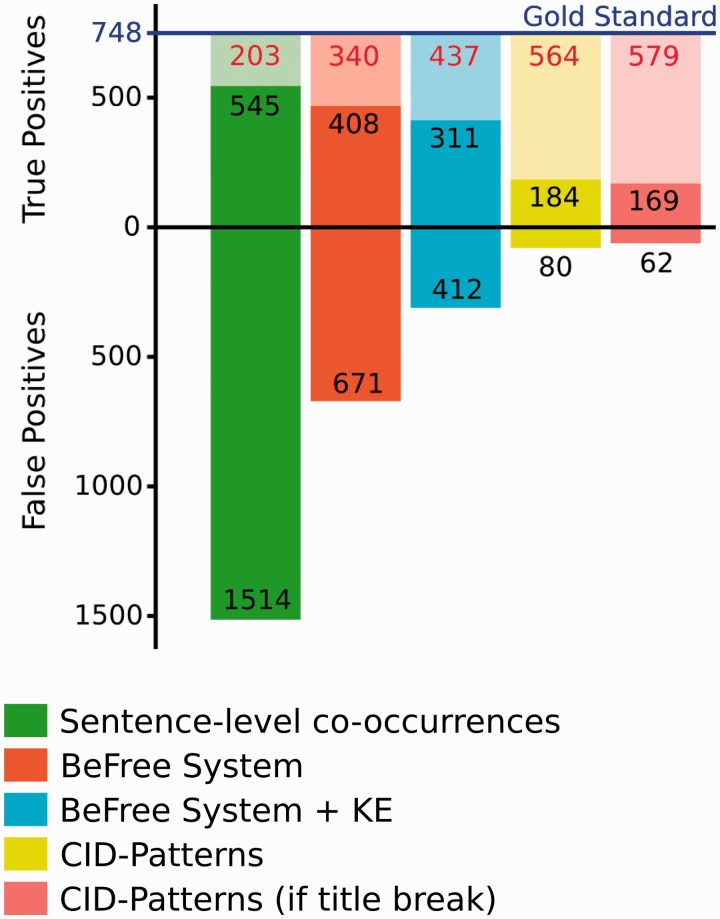
Number of CID-relations at the sentence level identified by our system in relation to the Gold standards. In each column, the lighter colors represent the fraction of FN.

We performed a more detailed analysis on the results obtained by the BeFree system and the CID Pattern approaches. We focused on the CID-relations stated at the sentence-level from the GS for this analysis. The results are shown in Figure 4. As expected, the identifications performed by the BeFree system improved the simple co-occurrence selection. Although the TPs results decreased slightly (consequently, decreasing the Recall), about ∼55% of the FPs were removed, increasing significantly the Precision. A similar trend was observed when the results from BeFree were filtered by using the EK approach (removing CID-relations annotated as therapeutic indications in the knowledge base). Although ∼40% of FPs were removed, the TPs were reduced in ∼20%. On the other hand, the CID Patterns approach achieved a high Precision, but the major problem of this approach is the high number of undetected CID-relations, achieving a very low Recall. It is interesting to note that, most CID-relations that follow a simple pattern can already identified in the title of the abstract (169/184, 91%).

**Figure 5.**
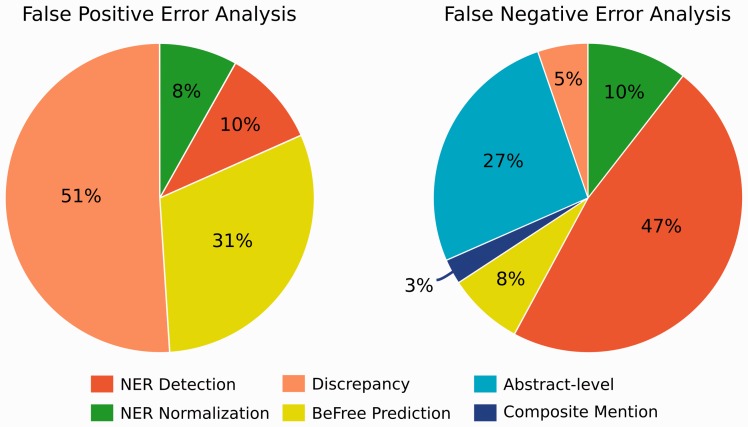
Percentages of the origin of the FP and FN reported by the BeFree system.

We then performed a detailed error analysis of the FP and FN results produced by BeFree system on a subset of abstracts (30 abstracts reporting FP and 30 reporting FN results). We identified 6 types of errors in our analysis. Table 3 shows a brief description and some examples for each type of error, and Figure 5 summarizes each error type in the FP and FN sets.

**Table 3.** Description and examples of each type of error identified

Type of Error	Description and examples
**NER Detection**	When the NER (DNorm or tmChem) did not detect a mention in the text.
	**Example** no. **1 (PMID:10764869)**‘**Tacrolimus** and other immunosuppressive agents may be associated with optic nerve **toxicity’**. ‘The GS relation is **Tacrolimus- optic nerve toxicity.** DNorm system did not properly detect ‘optic nerve toxicity’, but only ‘toxicity’. BeFree predicted the relation **Tacrolimus-Toxicity** as a CID-relation causing a FP and FN. **Example** no. **2 (PMID:10726030)** ‘However, additional important untoward effects of **heparin** therapy include **heparin**-induced **thrombocytopenia**, **heparin**-associated **osteoporosis**, **eosinophilia**, skin reactions, **allergic reactions** other than **thrombocytopenia**, **alopecia**, transaminasemia, **hyperkalemia**, **hypoaldosteronism**, and **priapism**’. The ‘skin reactions’ mention was not detected causing a FN. The remaining CID-relations were properly detected. As additional comment, ‘transaminasemia’ is a SE not included in the GS (also, it is not detected by DNorm), in our opinion **Heparin-Transaminasemia** should be annotated as a CID-relation. **Example** no. **3 (PMID:11366874)** ‘A group of doctors in Boston warn that the protease inhibitor Viracept may cause an irregular heart beat, known as **bradycardia**, in people with HIV’. In this case, tmChem did not detect ‘Viracept’ as a drug causing a FN.
**NER normalization**	When the NER did not correctly normalized a mention in the text.
	**Example** no. **4 (PMID:11026989)** ‘The effect of **pupil dilation** with **tropicamide** on vision and driving simulator performance’. In this example, ‘pupil dilation’ was normalized with MeSH:D002311, but in the GS the mentions was annotated with MeSH:D015878, causing a FP and FN, because BeFree predicted the entities as a CID-relation. **Example** no. **5 (PMID:10933650)** ‘Histopathological examination of the kidney showed **tubular necrosis** in **D-AmB**-treated rats but no change in NS-718-treated rats’. The entity ‘D-Amb’ was not normalized by the tmChem (in the GS was identified as MeSH:C059765), resulting in a FN.
**BeFree identification**	The BeFree system identified incorrectly a CID-Relation.
	**Example** no. **6 (PMID:11385188)** ‘Prevalence of **heart disease** in asymptomatic chronic **cocaine** users’. ‘We conclude that coronary artery or **myocardial disease** is common (38%) in young asymptomatic chronic **cocaine** users’. In the first sentence, the BeFree system identified the ‘Cocaine’- ‘Heart Disease’ association as a true CID-relation, causing a FP. Although in the second sentence, the BeFree system reported as a false CID-relation the ‘Cocaine-Myocardial Disease’ association, causing a FN. **Example** no. **7 (PMID:10462057)** ‘No **necrosis** was observed in cells treated with **NCQ436** but **NCQ344** had a biphasic effect in both cell types, inducing apoptosis at lower concentrations and necrosis at higher concentrations’. ‘We propose that **remoxipride** and **benzene** may induce **aplastic anemia** via production of similar reactive metabolites and that the ability of **NCQ436** and **NCQ344** to induce apoptosis in HBMP cells may contribute to the mechanism underlying acquired **aplastic anemia** that has been associated with **remoxipride’**. In the first sentence, the BeFree system improperly reported the ‘NCQ436- Necrosis’ association as a true CID-relation, causing a FP. Also, in the second sentence, the BeFree system identified as a true CID-relation the ‘NCQ436/NCQ344-Aplastic Anemia’, causing two FPs. Example no. 8 (PMID:11439380) ‘**Thalidomide** neuropathy in patients treated for metastatic **prostate cancer’**. In this example, the BeFree system identified ‘Thalidomide-Prostate Cancer’ as a CID-relation, including a FP. Example no. 9 (PMID:10327032) ‘Risk of transient hyperammonemic **encephalopathy** in cancer patients who received continuous infusion of **5-fluorouracil** with the complication of **dehydration** and **infection’**. In this example, the BeFree system identified ‘5-Fluorouracil-Dehydration’ as a CID-relation, including a FP. As additional comment, the GS annotated ‘encephalopathy’ as a diseases, but under our criteria, the annotated disease mention should be ‘transient hyperammonemic encephalopathy’ or ‘hyperammonemic encephalopathy’.
**Composite mention**	When a single span refers to more than one concept.
	**Example** no. **10 (PMID:10401555)** ‘**Nociceptin**/orphanin FQ and **nocistatin** on learning and **memory impairment** induced by **scopolamine** in mice’.In this sentence, BeFree only predicted one association as CID-relation, the ‘Memory Impairment’-‘Scopolamine’ association. ‘Learning Impairment’ was not detected, because our workflow does not detect composite mentions, causing a FN.
**Abstract-level**	The CID-relation only happening at the abstract-level, so BeFree cannot identify it.
	**Example** no. **11 (PMID:11229942)** ‘**Ventricular arrhythmias** were primarily observed in the C+E group, in which four of eight dogs experienced **ventricular tachycardia’**. ‘Ventricular tachycardia’ was annotated as CID-relation with ‘Ethanol’ and ‘Cocaine’.
**Discrepancy**	This type of error is perhaps the most controversial, where we have indicated the possible inconsistencies between the GS and our criteria for CID-relations.
	**Example** no. **12 (PMID:10401555)** ‘On the other hand, **nocistatin** is recently isolated from the same precursor as **nociceptin** and blocks **nociceptin**-induced **allodynia** and **hyperalgesia’**. The BeFree system predicted ‘Nociceptin-Allodynia’ and ‘Nociceptin-Hyperalgesia’ as two CID-relations not included in the GS. **Example** no. **13 (PMID:10726030)** ‘**Bleeding** is the primary untoward effect of **heparin’**. ‘**Major bleeding** is of primary concern in patients receiving **heparin** therapy’. The BeFree system predicted ‘Bledding/Major bledding’-‘Heparine’ as a true CID-relation not included in the GS. **Example** no. **14 (PMID:11425091)** The incidence of **subependymal cysts** in infants exposed to **cocaine** prenatally was 44% (8 of 18) compared with 8% (8 of 99) in the unexposed group (*P* < 0.01). The GS annotated ‘subependymal’ and ‘cysts’ as two different diseases. Dnorm detected ‘subependymal cysts’ as an unique disease and the BeFree system predicted as CID-relation the association with ‘cocaine’, causing two FN and one FP. **Example** no. **15 (PMID:10713017)** ‘The use of **appetite suppressants** in Europe has been associated with the development of **primary pulmonary hypertension (PPH)’**. The BeFree sytems reported the ‘**appetite suppressants**’-‘**PPH’** as a CID-relation, but it was not annotated in the GS.

We show in bold the entities detected by the NER, and underlined the entities that were not detected.

As shown in Figure 5, most of the errors reported by the BeFree system were not related to the system itself. In the case of FP, most of the errors are due to discrepancies (51%), followed by BeFree identification errors. On the other side, NER errors, including entity detection and normalization, together with associations spanning several sentences were the main reasons of the reported FNs. 8% of FP were explained by BeFree identification errors. By inspecting the examples that caused erroneous identification by BeFree, we can identify the main problems of the method as: i) incorrect handling of negations, ii) long and complicated sentences with several potentially related entities, iii) incorrect distinction between therapeutic indication of the drug and SEs.

## Conclusion and future work

In this paper we have presented a new system to detect CID-relations leveraging on machine learning, rule-based approaches and background knowledge. We showed that the combination of these different approaches addresses an ambitious challenge as the BC5, achieving competitive results. Specifically, our system achieved the highest Recall in the BC5 challenge, with values of ∼79 and ∼63% on the BC5_D_ and BC5_E_, respectively, approaching the Recall upper limit of each of them (81 and 72% on the BC5_D_ and BC5_E_, respectively).

Availability of high quality corpora is a pre-requisite for supervised machine learning systems and generally requires considerable efforts to build them (25). We have presented a new corpus developed by a crowdsourcing approach. The BeFree system trained on the Crowd-CID corpus achieved high performance in the detection of CID-relations (82.03, 73.39 and 76.82% of Precision, Recall and *F*-score, respectively, evaluated by 10-fold cross-validation). These results suggest that crowdsourcing is a good alternative to develop a corpus on CID-relations in a fast and cheap way, which can be used to train a machine learning system (BeFree system) for the identification of CID-relations.

Regarding the performance of our system, the low Precision obtained could be explained by our approach to detect relations at the abstract level, which considers all possible co-occurrences and relies on background knowledge to remove FPs. The error analysis performed on the BeFree results allowed the identification of some of the weak points of the system, such as incorrect handling of negations, poor performance on long and complicated sentences with several potentially related entities, and incorrect distinction between therapeutic indication of the drug and SEs.

Future work includes the evaluation of syntactic dependency features in BeFree to improve the identification of CID-relations. In addition, methods for the identification of relations spanning several sentences such as anaphora resolution and the correct detection of composite mentions could be used to improve significantly the overall results. Finally, training the system to distinguish between therapeutic use and drug SEs could improve the results.

## Funding

This work was supported by grants from the National Institutes of Health (GM114833, GM089820, TR001114); the Instituto de Salud Carlos III-Fondo Europeo de Desarrollo Regional (PI13/00082 and CP10/00524 to A.B. and L.I.F.); the Innovative Medicines Initiative-Joint Undertaking (eTOX no. 115002, Open PHACTs no. 115191, EMIF no. 115372, iPiE no. 115735 to A.B. and L.I.F.), resources of which are composed of financial contributions from the European Union’s Seventh Framework Programme (FP7/2007–13) and European Federation of Pharmaceutical Industries and Associations; and the European Union Horizon 2020 Programme 2014–20 (MedBioinformatics no. 634143 and Elixir-Excelerate no. 676559 to A.B. and L.I.F.). The Research Programme on Biomedical Informatics (GRIB) is a node of the Spanish National Institute of Bioinformatics (INB).

*Conflict of interest*. None declared.
